# *In-situ* constructed Cu/CuNC interfaces for low-overpotential reduction of CO_2_ to ethanol

**DOI:** 10.1093/nsr/nwac248

**Published:** 2022-11-03

**Authors:** Yan Yang, Jiaju Fu, Yixin Ouyang, Tang Tang, Yun Zhang, Li-Rong Zheng, Qing-Hua Zhang, Xiao-Zhi Liu, Jinlan Wang, Jin-Song Hu

**Affiliations:** Beijing National Laboratory for Molecular Sciences (BNLMS), CAS Key Laboratory of Molecular Nanostructure and Nanotechnology, Institute of Chemistry, Chinese Academy of Sciences, Beijing 100190, China; Research Institute, Zhejiang Tiandi Environmental Protection Technology Co. Ltd, Hangzhou 310003, China; University of Chinese Academy of Sciences, Beijing 100049, China; Beijing National Laboratory for Molecular Sciences (BNLMS), CAS Key Laboratory of Molecular Nanostructure and Nanotechnology, Institute of Chemistry, Chinese Academy of Sciences, Beijing 100190, China; School of Physics, Southeast University, Nanjing 211189, China; Beijing National Laboratory for Molecular Sciences (BNLMS), CAS Key Laboratory of Molecular Nanostructure and Nanotechnology, Institute of Chemistry, Chinese Academy of Sciences, Beijing 100190, China; Institute for Advanced Study, Shenzhen University, Shenzhen 518060, China; Institute of High Energy Physics, Chinese Academy of Sciences, Beijing 100049, China; Institute of Physics, Chinese Academy of Sciences, Beijing 100190, China; Institute of Physics, Chinese Academy of Sciences, Beijing 100190, China; University of Chinese Academy of Sciences, Beijing 100049, China; School of Physics, Southeast University, Nanjing 211189, China; Beijing National Laboratory for Molecular Sciences (BNLMS), CAS Key Laboratory of Molecular Nanostructure and Nanotechnology, Institute of Chemistry, Chinese Academy of Sciences, Beijing 100190, China; University of Chinese Academy of Sciences, Beijing 100049, China

**Keywords:** Cu/CuNC interface, low overpotential, CO_2_ reduction, ethanol, electrocatalysis

## Abstract

Electrochemical CO_2_ reduction (ECR) to high-value multi-carbon (C_2+_) products is critical to sustainable energy conversion, yet the high energy barrier of C-C coupling causes catalysts to suffer high overpotential and low selectivity toward specific liquid C_2+_ products. Here, the electronically asymmetric Cu-Cu/Cu-N-C (Cu/CuNC) interface site is found, by theoretical calculations, to enhance the adsorption of *CO intermediates and decrease the reaction barrier of C-C coupling in ECR, enabling efficient C-C coupling at low overpotential. The catalyst consisting of high-density Cu/CuNC interface sites (noted as ER-Cu/CuNC) is then accordingly designed and constructed *in situ* on the high-loading Cu-N-C single atomic catalysts. Systematical experiments corroborate the theoretical prediction that the ER-Cu/CuNC boosts electrocatalytic CO_2_-to-ethanol conversion with a Faradaic efficiency toward C_2+_ of 60.3% (FE_ethanol_ of 55%) at a low overpotential of −0.35 V. These findings provide new insights and an attractive approach to creating electronically asymmetric dual sites for efficient conversion of CO_2_ to C_2+_ products.

## Teaser

Cu-Cu/Cu-N-C multiatomic sites are discovered and achieved to synergistically promote the electrochemical reduction of CO_2_ into ethanol at low overpotentials by effectively lowering the energy barrier of C-C coupling.

## INTRODUCTION

Renewable-energy-driven electrochemical CO_2_ reduction (ECR) to fuels and value-added chemicals offers a sustainable opportunity to mitigate the environmental crisis and achieve carbon neutrality [1–4]. With the advantages of high energy density and economic value, liquid-phase multi-carbon products (C_2+_ products, e.g. ethanol and acetate) are considered ideal products in the chemical industry [2]. To date, Cu-based materials are known to be the most promising catalysts for harvesting C_2+_ products with considerable performance [5]. However, the complicated mechanisms of the multi-step pathways and high energy barriers for ECR to C_2+_ products, especially the C-C coupling step, brings significant challenges to developing high-performance catalysts with well-defined active sites for selectively reducing CO_2_ into desired C_2+_ products [6].

Various Cu-based catalysts have been recently reported to achieve the high-efficiency production of C_2+_ products like ethylene at high potentials, such as over −1.0 V (all potentials hereafter are versus reversible hydrogen electrode (RHE) [5–7]). The corresponding theoretical studies demonstrated that the C-C coupling step of *CO and CO-derived intermediates (*CHO and *COH) in most pathways for overall ECR to C_2+_ products possessed the highest energy barrier [1,8,9]. Although applying more negative potentials could overcome the high energy barrier of C-C coupling and enhance the selectivity toward C_2+_ products, the activity of the competitive hydrogen evolution would also be enhanced simultaneously, and the total energy efficiency of the whole electrolyzer would be decreased [10]. Besides, the over-input energy would directly reduce CO_2_ into more thermally stable products (methane or ethylene) instead of the desired liquid C_2+_ products [5–7,11,12].

The key to boosting C-C coupling at low overpotentials is to decrease the kinetic energy barrier of the C-C coupling process. One efficient way is to construct the interfaces of Cu^δ+^ and Cu^0^ on the catalytic surface, which can decrease the energy of C-C coupling by promoting the adsorption and dimerization of CO intermediates, thus enhancing the activity of CO_2_-to-C_2+_ conversion [13]. Heteroatoms with high electronegativity like oxygen and halides, or electron-deficiency structures like boron, were applied to construct interfaces of Cu^δ+^ and Cu^0^ for promoting C-C coupling [11,14–16]. For example, Cuenya *et al*. conducted a plasma-activated Cu to promote the conversion of CO_2_ to C_2+_ products at −0.9 V [11]; Sargent *et al*. used positive-valence boron to regulate the ratio of Cu^δ+^ to Cu^0^ on the surface to enhance the selectivity toward C_2+_ products at −1.1 V [14]. However, current Cu catalysts with mix-valence interfaces usually comprise a mixture of Cu, Cu^+^ and even Cu^2+^ species and introduce diverse types of Cu sites on the catalytic surfaces, significantly lowering the selectivity toward the specific product [17]. Considering that the kinetic energy levels needed for producing each C_2+_ product are similar [18,19], advanced strategies for delicately constructing Cu-based catalysts with specific active sites are urgently needed to selectively convert CO_2_ into specific C_2+_ products.

Here, in order to develop high-performance ECR catalysts for producing desired C_2+_ products, we seek an approach to the catalyst design for decreasing the kinetic energy barrier of the C-C coupling. Based on a former reported C-C coupling pathway towards C_2+_ products [20], density functional theory (DFT) was applied to investigate the kinetics and thermodynamics of reactions at different interface sites and it was found that the electronically asymmetric structured interface sites that formed on the interface of Cu-Cu and Cu-N-C sites (Cu/CuNC) exhibit the low energy barrier of only 0.30 eV for the C-C coupling promoted by the delocalized electrons. More importantly, we experimentally verify this concept by constructing well-defined Cu/CuNC interface sites on a Cu nanoparticle catalyst (ER-Cu/CuNC), electrochemically reduced, *in situ*, from a high-loading Cu-N-C single atomic catalyst (SAC). Both *in-situ* and *ex-situ* characterizations were applied to unveil the formation process and detailed structures of the Cu/CuNC interface sites. The as-described ER-Cu/CuNC catalyst exhibited an outstanding ECR performance with 60.3% total Faradaic efficiency of C_2+_ products (FE_C2+_) and an ethanol selectivity (FE_ethanol_) of 55% at only −0.35 V. In contrast, the control catalysts, including similar Cu nanoparticles without Cu/CuNC interface sites, Cu-N-C SACs, and the physical combination of Cu nanoparticles and Cu-N-C SACs, all show a negligible ethanol yield. Together with the comparison with systematically designed control catalysts, these results suggest the critical role of Cu/CuNC interface sites in enhancing the conversion efficiency of ECR to C_2+_ products, providing new insights and a strategy for exploring highly efficient ECR electrocatalysts by creating interfaces with electronically asymmetric dual-site centers.

## RESULTS AND DISCUSSION

**Figure 1.**
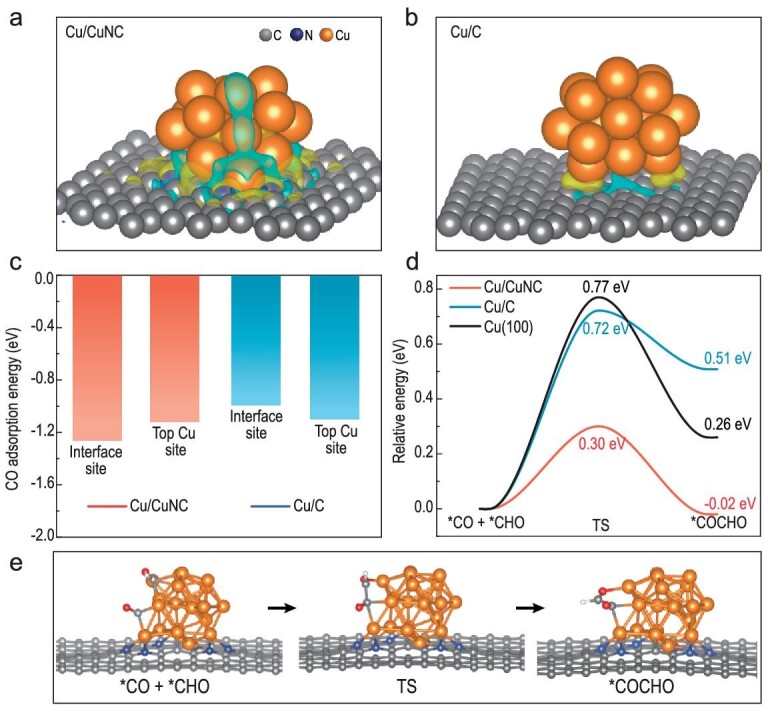
Theoretical studies. (a, b) Charge density difference map on different interface sites. The iso-surface level is 0.001 eV Å^−3^. Yellow and cyan refer to electron enrichment and depletion, respectively. (c) Calculated adsorption energies of CO on Cu_20_ clusters supported on N-doped porous carbon (Cu/CuNC) and graphene (Cu/C). (d) C-C coupling barriers on Cu/CuNC, Cu/C and Cu (100) surfaces. (e) C-C coupling process on Cu/CuNC.

DFT calculations were firstly applied to investigate the influence of the Cu/CuNC interface sites on the C-C coupling process. We constructed three model systems: Cu (100) single crystal, and Cu_20_ clusters supported on N-doped porous carbon and graphene, denoted as Cu (100), Cu/CuNC and Cu/C, respectively. As shown in the charge-density-difference maps of Fig. 1a and b, the Cu/CuNC structure exhibits more significant electron transfer at the interface sites than the Cu/C structure on the charge density difference maps. Furthermore, the asymmetric electron distribution at the Cu/CuNC interface sites demonstrates that the Cu^δ+^/Cu^0^ structure was established with Cu-N sites as positively charged (yellow area) Cu^δ+^, and the negatively charged (cyan area) top Cu atoms as Cu^0^ sites. Then, we calculated the adsorption energies of key intermediate *CO. Cu/CuNC and Cu/C models show a preferred *CO adsorption on the interface sites and the top Cu sites, respectively (Fig. 1c and Supplementary Fig. 1). It is thus reasonable to consider the Cu-Cu/Cu-N-C interface sites in Cu/CuNC and the top Cu sites in Cu-C as reaction centers. In addition, the reaction free energy calculations indicate that *CO is easily formed at the Cu/CuNC interface (Supplementary Fig. 2), which is highly favorable for the next C-C coupling step. The C-C coupling barriers are further calculated as shown in Fig. 1d. The formation of C-C bonds is the most critical step in the process of generating C_2+_ products, while it is also the most elusive [21–23]. Given the coordination saturation degree of carbon atoms in intermediates and the concentration of various intermediates on the catalyst surface, *CO-*CO, *CO-*CHO and *CO-*COH coupling are proposed as the three most dominant paths to produce multi-carbon products [23]. The calculated reaction energies show that the *CO-*CHO coupling is thermodynamically most favored among the three coupling paths (Supplementary Fig. 3), thus the barrier of *CO-*CHO coupling is used to represent the formation trends of C_2+_ products (Fig. 1e). It can be clearly seen that the Cu-Cu/Cu-N-C interface sites significantly decreased the reaction barrier of *CO-*CHO coupling (0.30 eV) compared to those on Cu/C (0.72 eV) and Cu (100) (0.77 eV) surface sites. Apart from the low kinetic barrier, the C-C coupling process on the Cu/CuNC interface sites is also thermodynamically more favorable. This suggested that C-C coupling is likely no longer the main obstacle to generating multi-carbon products, and the proton-coupled electron transfer (PCET) step, such as *CO to *CHO, became the rate-limiting step. The strong dependence of the PCET step on the electrode potentials could lead to the distribution of reduction products being very sensitive to the applied potential [19].

**Figure 2.**
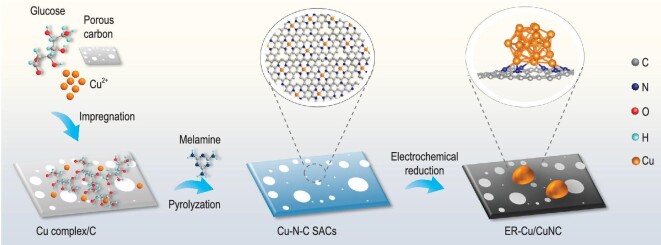
Schematic illustration of the preparation of ER-Cu/CuNC.

**Figure 3.**
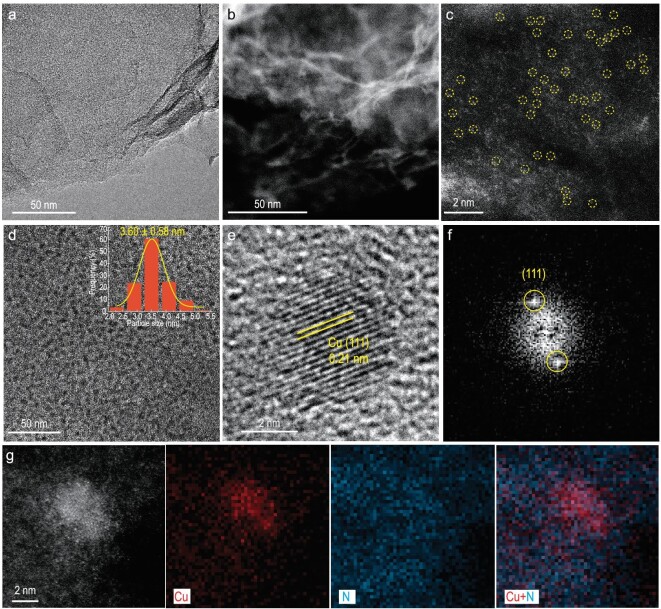
Morphology characterizations. (a) TEM image. (b) STEM image. (c) HAADF-AC-STEM image for Cu-N-C SAC precursor. (d–g) TEM image and the particle size distribution (d), high-resolution transmission electron microscopic image (HRTEM) (e), corresponding FFT pattern (f) and HAADF-AC-STEM image and EELS mapping images of Cu, N and overlaid Cu and N (g) for ER-Cu/CuNC.

**Figure 4.**
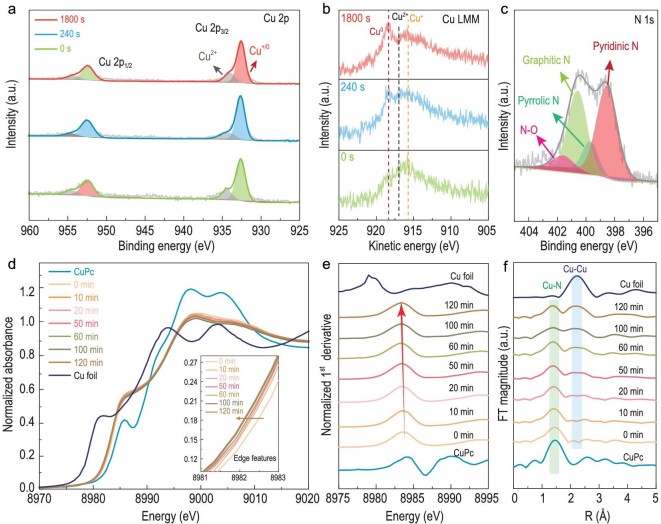
Structural characterizations. (a) Cu 2p XPS spectra. (b) Cu LMM Auger spectra under different Ar cluster etching time. (c) N1s XPS spectra of ER-Cu/CuNC. (d–f) The operando Cu K-edge XANES spectra (d), the corresponding first derivative spectra (e) and the operando Cu K-edge EXAFS spectra (f) for the Cu/CuNC sample under different electrochemical reduction times at −0.30 V.

To construct the Cu/CuNC structure experimentally, we developed an electroreduction method to form the Cu/CuNC interface sites *in situ* from the high-density N-anchored single-site Cu sample (Cu-N-C). As illustrated in Fig. 2, the as-described ER-Cu/CuNC catalyst with abundant Cu/CuNC interface sites was constructed by electrochemically reducing a Cu-N-C SAC supported on the three-dimensional (3D) honeycomb-like porous carbon (Supplementary Fig. 4a), which was prepared according to our previous report [24]. The structure of the Cu-N-C precursor was first characterized by transmission electron microscopy (TEM) and scanning transmission electron microscopy (STEM) (Fig. 3a and b and Supplementary Fig. 4b) with energy-dispersive X-ray spectroscopy (EDS) analysis (Supplementary Fig. 5). No particles were observed on the 3D carbon support with the uniformly elemental distribution of Cu, suggesting the atomic dispersion of Cu. No Cu peaks appeared in the powder X-ray diffraction (XRD) patterns (Supplementary Fig. 4c), and atomic-dispersed Cu observed by the atomic-resolution high-angle annular dark-field aberration-corrected scanning transmission electron microscopy (HAADF-AC-STEM, Fig. 3c) corroborated the successful synthesis of the Cu-N-C SAC. For the ER-Cu/CuNC catalyst, Cu nanoparticles with a diameter of 3.60 ± 0.58 nm that were uniformly dispersed on the carbon support were obtained after the *in-situ* electroreduction process (Fig. 3d). A lattice space of 0.21 nm was observed on the high-resolution TEM image with fast Fourier transform (FFT) analysis (Fig. 3e and f), corresponding to the (111) facet of Cu. The ER-Cu/CuNC catalyst (Supplementary Fig. 6) presented a typical pattern of porous carbon, representing the low content and the ultrafine crystal size of the *in-situ*-formed Cu nanoparticles [3]. More importantly, the combined HAADF-AC-STEM and high-resolution electron energy loss spectroscopic (EELS) images in Fig. 3g exhibit the *in-situ*-grown Cu nanoparticle surrounded by abundant N, implying the formation of the Cu-Cu/Cu-N interface sites. For comparison, the samples with only Cu-Cu sites (Cu nanoparticles with a diameter of 5.18 ± 0.83 nm, Cu/C, Supplementary Fig. 7), Cu-N sites (copper phthalocyanine with well-defined CuN_4_ sites on carbon support, CuPc/C, Supplementary Figs 8–10), a physical combination of Cu-N and Cu-Cu sites (CuPc molecules supported on the above Cu/C, CuPc-Cu/C, Supplementary Fig. 11) and Cu nanoparticles with a diameter of 4.72 ± 0.93 nm supported on N-doped porous carbon (Cu/NC, Supplementary Figs 12 and 13) were also prepared on the same porous carbon support. To avoid the interference of metal loading on activity, the feeding of inorganic copper sources was kept the same (Supplementary Table 1). The X-ray photoelectron spectra (XPS) revealed the surface states of as-described samples. The survey spectrum verified the presence of C, N, O and Cu elements on the Cu-N-C SAC precursor (Supplementary Fig. 14a). The high-resolution N 1s spectrum (Supplementary Fig. 14b) showed the major N species on the Cu-N-C surface was pyridinic N. Further, the combined Cu 2p spectra with Auger spectrum showed that the Cu atoms were positively charged on the Cu-N-C surface (Supplementary Fig. 14c and d), indicating a typical state of the single-site Cu structure. For ER-Cu/CuNC, the fresh-made catalyst was directly dried under an N_2_ atmosphere and immediately transferred for Ar cluster sputtering to avoid the oxidation of copper. As shown in Fig. 4a and b, the primary Cu state on the surface of the catalyst remained in Cu^0^/Cu^+^ at 932.5 eV during the formation of the ER-Cu/CuNC. The Auger spectra of Cu LMM further revealed the coexistence of zero-valent Cu (918.5 eV) and positively charged Cu on the surface of the ER-Cu/CuNC sample [25,26]. The existence of positively charged Cu species could be attributed to the strong interaction from the surface Cu-N structures shown in Fig. 4c [27].

The operando X-ray absorption near-edge structure (XANES) measurement was applied at Cu K-edge to further investigate the evolution of the Cu chemical state and local structure during the formation of the ER-Cu/CuNC catalyst. As shown in Fig. 4d and e, compared with CuPc, the time-dependent negative shift of the adsorption edge represented the decreasing average valence of Cu, demonstrating the formation of Cu^0^ species from the reduction of positively charged Cu [27,28]. Moreover, consistent with *in-situ* XANES spectra, the extended X-ray absorption fine structure (EXAFS) analysis shown in Fig. 4f revealed the formation of Cu-Cu bonds located at ∼2.2 Å on the horizontal axis during the electrochemical treatment. By contrast, the simultaneous decreasing of Cu-N bonds located at ∼1.5 Å on the horizontal axis suggested the *in-situ* growth of Cu nanoparticles from the N coordinated single-atomic Cu. Combined with the above EELS mapping and XPS results, these results suggest the formation of the Cu-Cu/Cu-N interface sites from Cu-N-C SACs in the ER-Cu/CuNC catalyst.

**Figure 5.**
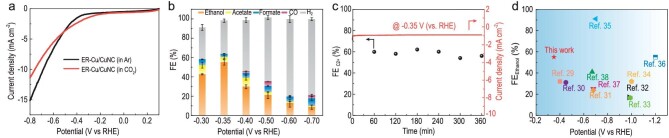
ECR performance of ER-Cu/CuNC in H-cells. (a) Linear sweep voltammetric (LSV) curves performed in Ar-saturated and CO_2_-saturated 0.1 M KHCO_3_ solution. (b) Potential-dependent Faradaic efficiencies (FEs) for different products. (c) Long-term durability of ER-Cu/CuNC for ECR operated at -0.35 V. (d) Comparison of applied potential and FE_ethanol_ for ER-Cu/CuNC and other previously reported Cu-based catalysts with FE_C2+_ above 50%.

The ECR performances of as-prepared catalysts were evaluated in an H-type cell using 0.1 M KHCO_3_ aqueous solution as electrolytes (please see details in Methods and the Supplementary Data). As shown in the linear sweep voltammetric curves (Fig. 5a), the larger current density before −0.5 V in CO_2_-saturated electrolytes than in Ar suggests higher ECR activity than hydrogen evolution at low overpotentials on the ER-Cu/CuNC catalyst. Then, the potential-dependent selectivity for ECR products of the ER-Cu/CuNC catalysts was analyzed in the range of −0.30 to −0.70 V. As shown in Fig. 5b and Supplementary Fig. 15, the products mostly contained H_2_ and CO in the gaseous phase, and ethanol, acetate and formate in the liquid phase, respectively. The ER-Cu/CuNC catalyst exhibited excellent selectivity toward C_2+_ products at low overpotentials and achieved a maximal FE_ethanol_ of 55% and FE_C2+_ products of 60.3% with a total current density of 0.98 mA m^−2^ at −0.35 V. In addition, the maximal FE ratio of C_2+_/C_1_ could reach 17.2 at −0.35 V, indicating a high C-C coupling efficiency (Supplementary Fig. 16). No appreciable decays of current density and FE_C2+_ (Fig. 5c), as well as no structural changes (Supplementary Fig. 17) after a 6 h continuous test at −0.35 V were observed, indicating the excellent stability of the ER-Cu/CuNC catalyst. The comparison of applied potential and FE_ethanol_ between this work and other previously reported Cu-based catalysts with FE_C2+_ above 50% in Fig. 5d [29–38] (please see detailed information in Supplementary Table 2), suggests that the construction of Cu-Cu/Cu-N interface sites with the asymmetric electronic structure center is an efficient strategy to develop C_2+_-selective electrocatalysts at low overpotential.

**Figure 6.**
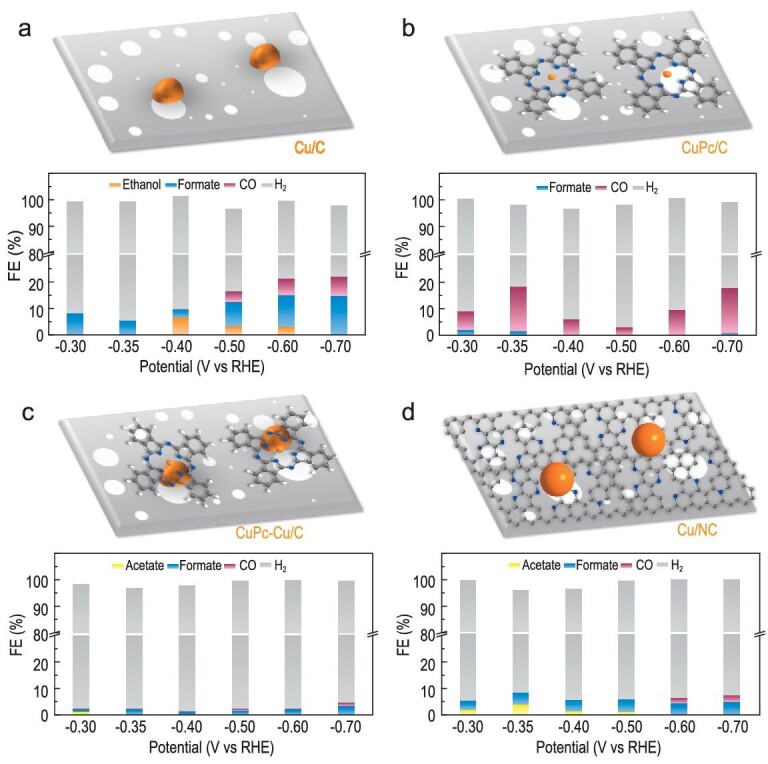
Potential-dependent FEs for ECR of control catalysts in H-cells: (a) Cu/C, (b) CuPc/C, (c) CuPc-Cu/C and (d) Cu/NC.

To get further insights into the improved C-C coupling on the ER-Cu/CuNC catalyst, a series of control catalysts were systematically designed and evaluated for ECR under the same conditions, including similar Cu nanoparticles without Cu-N-C sites (Cu/C, Fig. 6a and Supplementary Fig. 18), well-defined molecular model Cu-N-C sites without Cu nanoparticles (CuPc/C, Fig. 6b and Supplementary Fig. 19), the physical combination of Cu nanoparticles and molecular CuPc without the formation of the Cu/CuNC interface sites (CuPc-Cu/C, Fig. 6c and Supplementary Fig. 20) and Cu nanoparticles on N-doped carbon support without the interface sites of Cu/CuNC (Cu/NC, Fig. 6d and Supplementary Fig. 21). Both Cu/C and CuPc/C samples delivered low-efficiency C_2+_ products, indicating the sole Cu-Cu or Cu-N_4_ sites could hardly perform the C-C coupling at low potential. Furthermore, the low FE_C2+_ obtained on the CuPc-Cu/C and Cu/NC samples suggests the necessity of the tightly combined high-density Cu-Cu/Cu-N interface sites for forming C_2+_ products. The real-time ECR activity during the ER-Cu/CuNC formation is provided in Supplementary Fig. 22. The increase in FE_C2+_ during the formation of Cu-Cu/Cu-N interface sites (dependent on the electrochemical reduction time) suggests that the enhanced C-C coupling activity was from the *in-situ*-formed Cu-Cu/Cu-N interface sites. The FE of <100% was due to the ongoing *in-situ* reduction of Cu-N-C to Cu nanoparticles. The above results support that the excellent FE_C2+_ at low overpotential on the ER-Cu/CuNC catalyst should be attributed to the integrated Cu-Cu and Cu-N interface sites as predicted by the DFT calculations in Fig. 1. Besides, it is noted that the ER-Cu/CuNC also delivered much higher FE_C2+_ at higher potentials than all controls without such interface sites, which is to be expected with boosted C-C coupling. The decrease of FE_C2+_ with the increase of potentials could be attributed to the limited mass transfer of CO_2_ in the H-type cell, which could be further improved by the smart design of advanced electrolysis devices and the inhibition of H_2_ evolution.

## CONCLUSION

In conclusion, with the assistance of DFT calculations, we successfully constructed Cu/CuNC interface sites for high-efficiency CO_2_ conversion to C_2+_ products at low overpotentials by forming ER-Cu/CuNC electrocatalysts via *in-situ* electroreduction of the Cu-N-C SAC. The EELS, XPS and *in-situ* X-ray absorption spectra (XAS) investigations confirmed the formation of Cu-Cu/Cu-N interface sites. The resulting ER-Cu/CuNC electrocatalyst exhibited an excellent C_2+_ selectivity up to 60.3% with an FE_ethanol_ of 55% at a low potential of −0.35 V, standing out from the Cu-based ECR electrocatalysts for C_2+_ products. Systematically designed control experiments revealed that such a superior ECR performance at low potential should be ascribed to significantly enhanced electrocatalytic C-C coupling on Cu-Cu/Cu-N interface sites. DFT calculations showed that the Cu/CuNC interface site with an asymmetric electronic structure center was favorable for promoting the adsorption strength of the *CO intermediate and decreasing the reaction barrier of C-C coupling compared to those on Cu-Cu sites. These findings suggest an attractive strategy to promote CO_2_-to-C_2+_ conversion at low potential via designing tightly compounded interface sites and decreasing the energy barrier of the C-C coupling process, providing new insights into advanced catalyst design for producing specific products in ECR and other electrocatalytic reactions.

## METHODS

### DFT calculations

All DFT calculations were performed using the Vienna ab initio simulation package (VASP) with the Perdew–Burke–Ernzerhof (PBE) exchange-correlation functional [39–42]. In structural relaxation, the total energy and the force on each relaxed atom were converged to 10^−4^ eV and 0.02 eV Å^−1^, respectively. The plane-wave cutoff energy was 450 eV, and a k-mesh of 3 × 3 × 1 was adopted to sample the Brillouin zone. The van der Waals interactions were described by the density functional dispersion correction (DFT-D3). The climbing-image nudged elastic band (CI-NEB) method was used to locate the minimum-energy path [43,44]. Four images are uniformly distributed along the diffusion path connecting the initial and final states during the NEB calculation. The configurations of initial states and final states were fully relaxed.

### Materials

Potassium citrate monohydrate (K_3_C_6_H_5_O_7_·H_2_O, 99+%, Alfa Aesar Co., Ltd.), copper nitrate trihydrate (Cu (NO_3_)_2_·3H_2_O, 99%, Alfa Aesar Co., Ltd.), α-D-glucose (C_6_H_12_O_6_, 99%, Alfa Aesar Co., Ltd.), melamine (C_3_H_6_N_6_, 99%, Alfa Aesar Co., Ltd.), copper phthalocyanine (CuPc, Alfa Aesar Co., Ltd.), potassium bicarbonate (KHCO_3_, 99.7%–100.5%, Alfa Aesar Co., Ltd.). Sulfuric acid (H_2_SO_4_, 95%–98%, Sinopharm Chemical Reagent Co. Ltd.), N, N-dimethylformamide (DMF, 99.9%, Sinopharm Chemical Reagent Co. Ltd.).

### Preparation of porous carbon

Porous carbon was prepared according to our previous report [24].

### Preparation of Cu-N-C

Typically, 1.0 mmol of Cu(NO_3_)_2_·3H_2_O was first dissolved in 5 mL of deionized water. Then, 1.2 g of α-D-glucose and 60 mg of porous carbon were added to the above solution and sonicated for 30 min, followed by a 12 h store. The precipitates were collected by a centrifuge, dried overnight and then grounded by melamine with a mass ratio of 1:5. The obtained powder went through a pyrolyzation at 800°C for 2 h under an Ar atmosphere.

### Preparation of ER-Cu/CuNC

The ER-Cu/CuNC was prepared via the *in-situ* electrochemical reduction process of the as-prepared Cu-N-C. The Cu-N-C sample was firstly loaded on the working electrode following the process of working electrode preparation, then electrochemically reduced at -0.30 V vs. RHE for 2 h in 0.1 M KHCO_3_ electrolytes under CO_2_ reduction conditions to form the ER-Cu/CuNC catalysts. The as-prepared ER-Cu/CuNC catalysts were directly applied to the ECR performance test without further processing.

### Electrochemical measurements

All electrochemical measurements were conducted on a CHI660E electrochemical workstation in a standard three-electrode system with an H-cell configuration. The as-prepared electrode (please see details in the Supplementary Data) served as the working electrode, the Ag/AgCl electrode with saturated KCl solution served as the reference electrode, and the graphite rod served as the counter electrode. The anode and cathode compartments contained 15 mL 0.1 M KHCO_3_ aqueous electrolyte with a headspace of 10 mL, separated by a Nafion-117 proton exchange membrane.

## Supplementary Material

nwac248_Supplemental_File
