# Supplementary material for: In-situ constructed Cu/CuNC interfaces for low-overpotential reduction of CO2 to ethanol
Source: Natl Sci Rev. 2022 Nov 3;10(4):nwac248. doi: 10.1093/nsr/nwac248 (PMC10171628; doi:10.1093/nsr/nwac248)
Supplement: nwac248_Supplemental_File [file nwac248_supplemental_file.docx]

**In-situ Constructed Cu/CuNC Interfaces for Low-Overpotential Reduction of CO_2_ to Ethanol**

Yan Yang^1,6,7,†^, Jiaju Fu^1,†^, Yixin Ouyang^2,†^, Tang Tang^1^, Yun Zhang^3^, Li-Rong Zheng^4^, Qing-Hua Zhang^5^, Xiao-Zhi Liu^5,7^, Jinlan Wang^2,*^ & Jin-Song Hu^1,7,*^

^1^ Beijing National Laboratory for Molecular Sciences (BNLMS), CAS Key Laboratory of Molecular Nanostructure and Nanotechnology, Institute of Chemistry, Chinese Academy of Sciences, Beijing 100190, China.

^2^ School of Physics, Southeast University, Nanjing 211189, China.

^3^ Institute for Advanced Study, Shenzhen University, Shenzhen 518060, China.

^4^ Institute of High Energy Physics, Chinese Academy of Sciences, Beijing 100049, China.

^5^ Institute of Physics, Chinese Academy of Sciences, Beijing 100190, China.

^6^ Zhejiang Tiandi Environmental Protection Technology Co., Ltd, Hangzhou 310003, China.

^7^ University of Chinese Academy of Sciences, Beijing 100049, China.

These authors contributed equally: Yan Yang, Jiaju Fu, Yixin Ouyang

^*^Corresponding author. E-mail: [jlwang@seu.edu.cn](mailto:jlwang@seu.edu.cn); [hujs@iccas.ac.cn](mailto:hujs@iccas.ac.cn)

**Experimental Details**

**Electrochemical measurements.**

All electrochemical measurements were conducted on a CHI660E electrochemical workstation in a standard three-electrode system with an H-cell configuration. The as-prepared electrode was served as the working electrode, Ag/AgCl electrode with saturated KCl solution was served as the reference electrode, and the graphite rod was served as the counter electrode. The anode and cathode compartments contained 15 mL 0.1 M KHCO_3_ aqueous electrolyte (pH = 6.8) with a headspace of 10 mL, separated by a Nafion-117 proton exchange membrane (Alfa Aesar Co., Ltd.). Before the measurement, the high-purity CO_2_ was passed into the working electrode compartment for at least 30 min to make it pre-saturated. During measurement, the flow rate of CO_2_ blowing into the electrolyte was fixed to 10 mL min^-1^. The linear sweep voltammetry (LSV) was performed in CO_2_-saturated and Ar-saturated 0.1 M KHCO_3_ aqueous solution from 0.30 V to -0.80 V at a scan rate of 5 mV s^-1^. All potentials mentioned in this work were converted to the values vs. reversible hydrogen electrode (RHE) followed by the Nernst equation (1):

*E* (*vs* RHE) = *E* (*vs* Ag/AgCl) +0.198+0.059 × pH.

The Faradaic efficiency (FE) of the specific products was calculated by the following equation (2):

FE_i_ = *Q*_i_/*Q*_total_ = z × n_i_ × F/*Q*_total_

i: the specific products including CO, H_2,_ formate, ethanol, and acetate;

*Q*_i_: the partial charge used for the generation of specific products, C;

*Q*_total_: the total charge passed, C;

z: the number of transferred electrons for CO_2_-to-CO/formate conversion and H_2_O-H_2_ reduction, which is two for CO, formate, and H_2_; the number of transferred electrons for CO_2_-to-ethanol/ acetate conversion, which is twelve for ethanol and acetate;

n_i_: the number of moles for specific products;

F: Faradaic constant, which is 96,485 C mol^–1^;

All current densities (mA cm^-2^) mentioned in this article were normalized to the electrode geometrical area. The partial current density towards the specific product was calculated by the following equation (3):

*j*_i_=*j*_total_ × FE_i_

Where *j*_total_ is referred to the average total current density during whole electrocatalysis.

The gas chromatography (GC, Agilent Technologies 7890B) was employed to detect gaseous products generated from the cathode compartment. ^1^H Nuclear magnetic resonance (NMR, Bruker AVANCE 600) was employed to analyze the liquid products containing the mixture of 500 μL electrolyte after electrocatalysis, 100 μL of D_2_O, and 100 μL of 7.0 mM of dimethyl sulphoxide solution (DMSO, internal standard).

**The preparation of the working electrode.**

Typically, the mixed solution contained 2 mg of catalyst powder, 760 μL of ethanol, and 40 μL of 0.5 wt.% Nafion solution was sonicated for 30 min to form a uniform catalyst ink. The catalyst ink (400 μL) was then uniformly dropped onto the carbon cloth (CC), giving a catalyst loading of 1.0 mg cm^−2^. The working electrode was naturally dried for the following electrochemical test.

**Characterizations.**

Powder X-ray diffraction (XRD) pattern was performed on a Rigaku D/Max-2500 diffractometer using a Cu Kα1 radiation (λ = 1.54 Å). Scanning electron microscopic images (SEM) were obtained on a Hitachi scanning electron microscope (S-4800, Japan). Transmission electron microscopic (TEM) images were collected by the JEM-2100F microscope (JEOL, Tokyo, Japan) equipped with an EDS detector (Oxford Instrument, UK), working at an acceleration voltage of 200 kV. STEM images were attained on a JEOL ARM200F (JEOL, Tokyo, Japan) STEM under 200 kV with a cold filed-emission gun and double hexapole Cs correctors (CEOS GmbH, Heidelberg, Germany). Electron energy loss spectroscopy (EELS) data were obtained with a multi-scan charge-coupled device (CCD) camera (Gatan Quantum Model 965, Gatan Inc.). X-ray photoelectron spectroscopy (XPS) analysis was performed on an ESCALab220i-XL electron spectrometer (VG Scientific, UK) using a monochromatic Al Kα radiation as the X-ray source. Time-dependent *in-situ* X-ray absorption spectroscopy (XAS) results of Cu K-edge were obtained on the XAFS station of the 3W1 beamline of the Beijing Synchrotron Radiation Facility (BSRF) using a Si (111) double crystal monochromator. The *in-situ* ECR tests were conducted in a specifically-designed spectro-electrochemical cell sealed by Kapton films, and the 0.1 M KHCO_3_ aqueous electrolyte was added in the confined cathode chamber with continuously bubbled CO_2_. The time-dependent *in-situ* XAFS experiments were performed during chronoamperometry processes at -0.30 V, with the spectrum collected every 10 min.

**Preparation of Cu/C.**

Typically, 1mmol of Cu(NO_3_)_2_·3H_2_O and 60 mg of porous carbon were first dissolved in 5 mL of deionized water and sonicated for 30 min, followed by a 12 h store. The precipitates were collected by centrifuged and dried at 60 ^o^C overnight, followed by a pyrolyzation at 200 °C for two hours under the 10% H_2_/Ar atmosphere.

**Preparation of CuPc/C.**

Typically, 7.5 mg of CuPc was first dissolved in 60 mL of DMF and sonicated for 30 min. Then, 60 mg of porous carbon were added to the above solution and sonicated for 60 min, followed by a 24 h store. The precipitates washed several times and dried in a vacuum oven at 100 ^o^C.

**Preparation of CuPc-Cu/C.**

The control sample of CuPc-Cu/C was prepared via the same procedures as those for CuPc/C except for using Cu/C instead of the porous carbon.

**Preparation of** **Cu/NC.**

The porous carbon was firstly grounded by melamine with a mass ratio of 1:5. Then the obtained power was pyrolyzed at 800 °C for two hours under the Ar atmosphere to attain N-doped porous carbon. The Cu/NC was prepared via the same procedures as those for Cu/C except for using N-doped porous carbon instead of porous carbon.

**Supplementary figures**

**
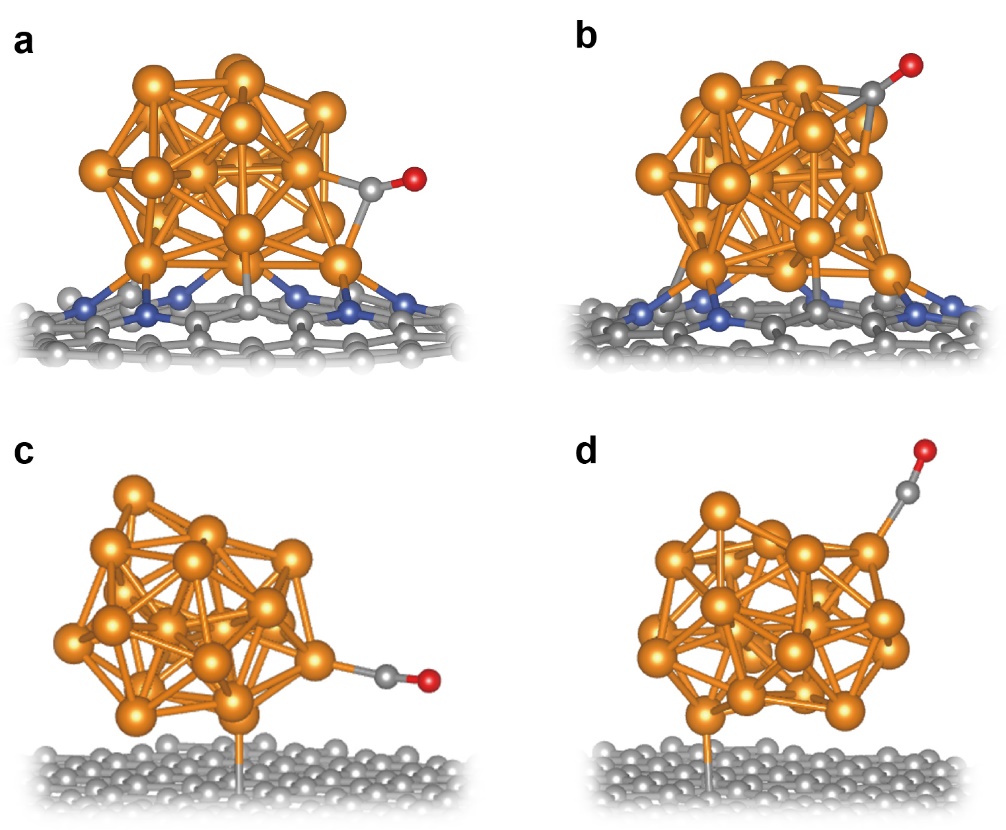
**

**Supplementary Fig. 1** *CO adsorption model on different sites of Cu/CuNC (a-b) and Cu/C (c-d).


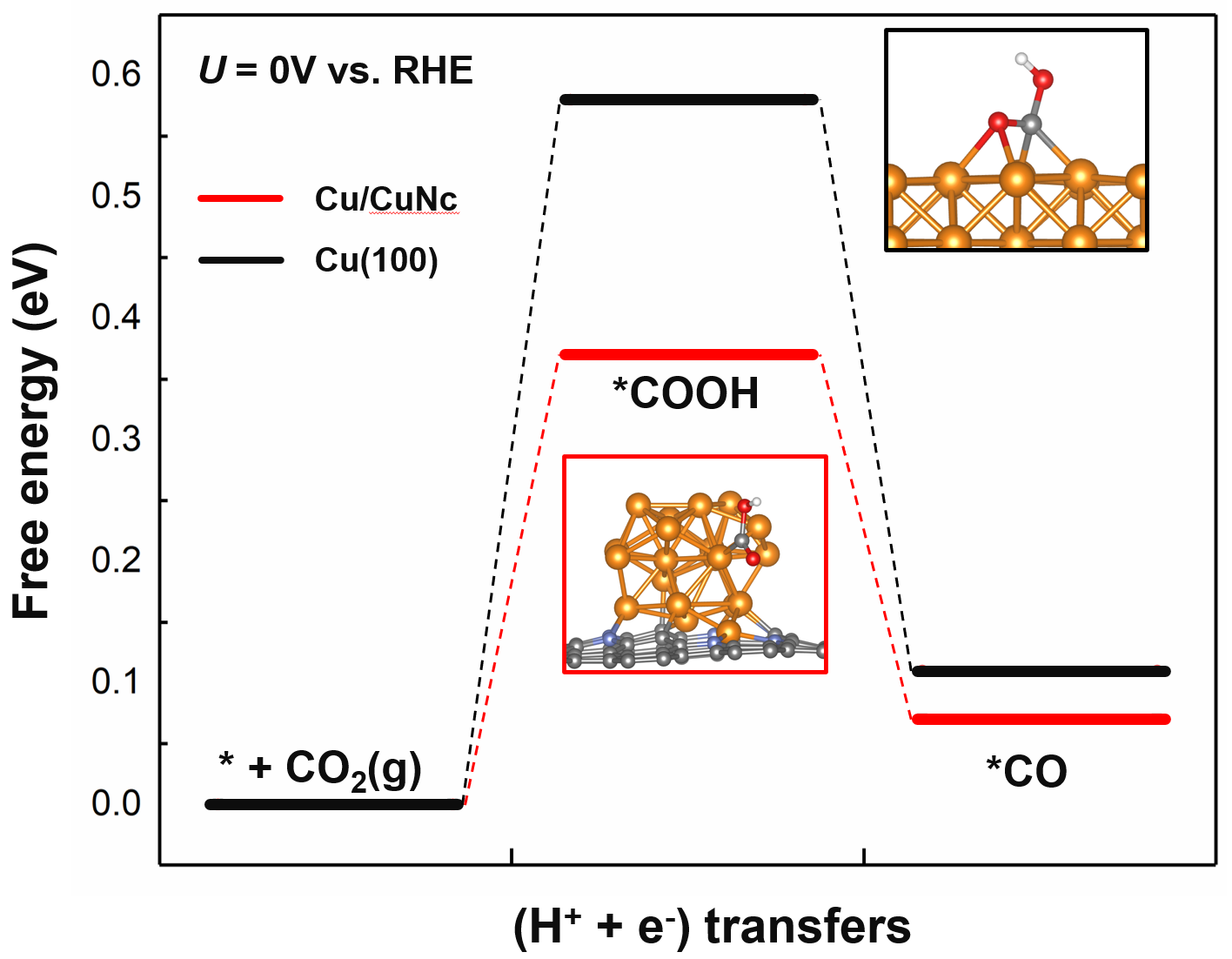


**Supplementary Fig. 2** Free energy diagram of CO_2_ reduction to *CO on Cu/CuNC and Cu(100) surface. Atomic configurations are shown as framed insets.


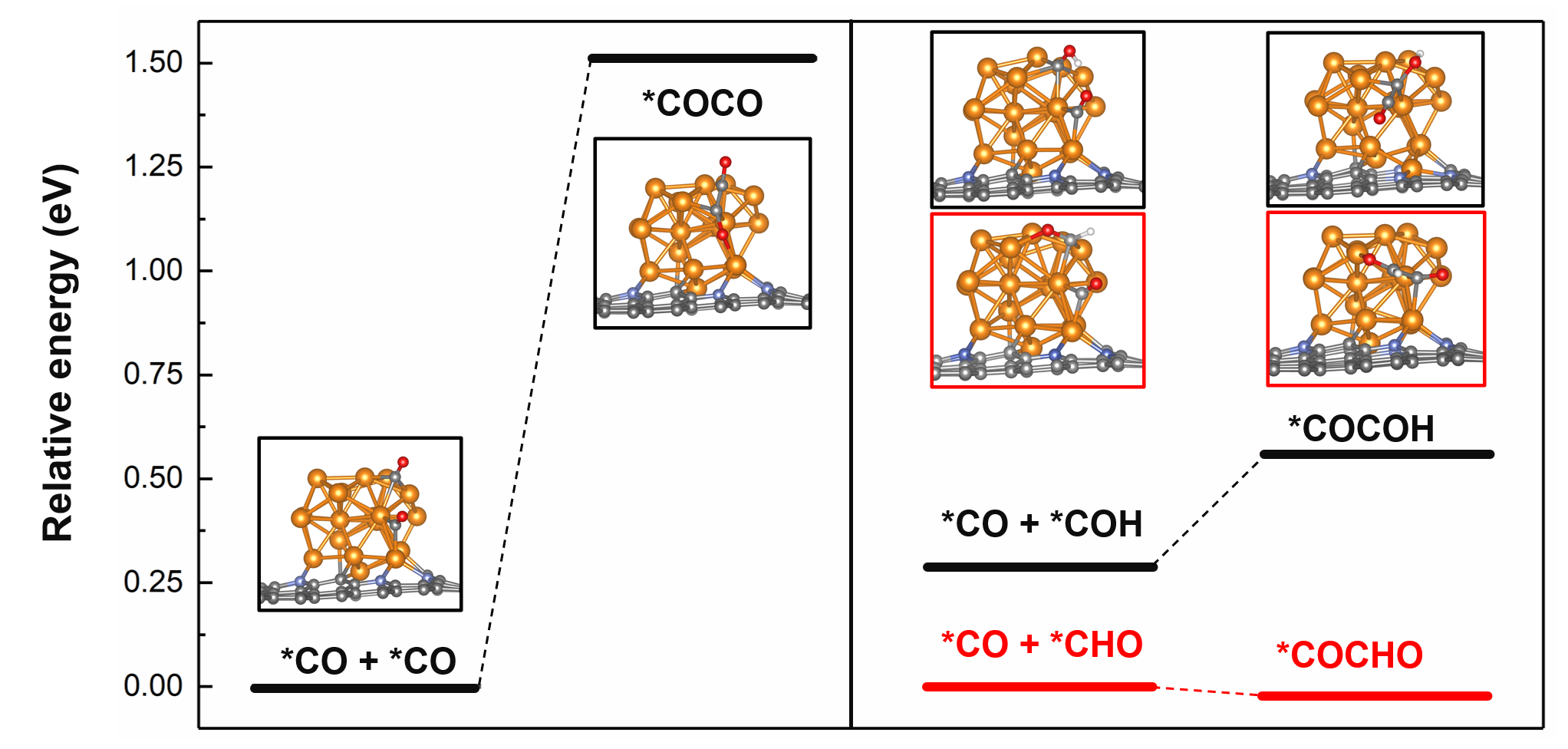


**Supplementary Fig. 3** Reaction energy diagram of *CO-*CO, *CO-*CHO and *CO-*COH coupling on Cu/CuNC interface. Atomic configurations are shown as framed insets.


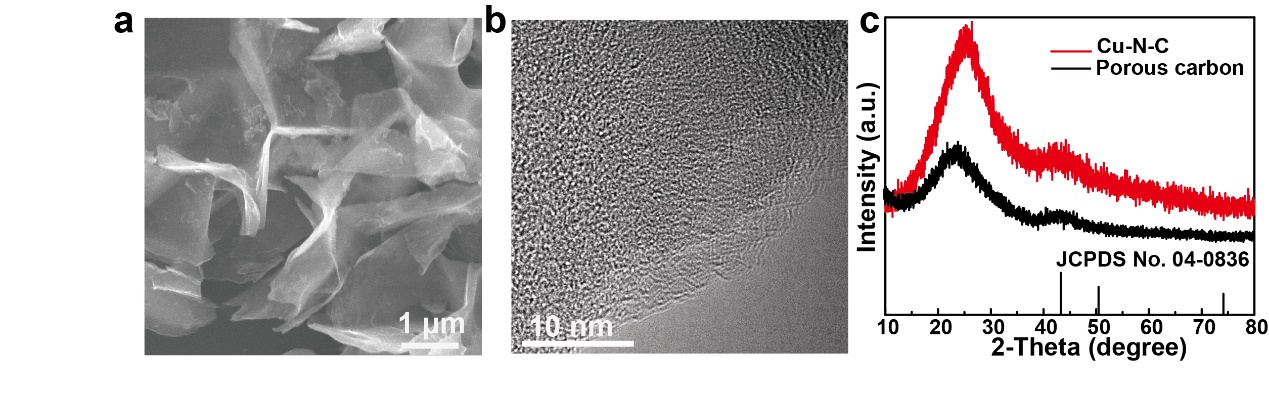


**Supplementary Fig. 4** (a) SEM image of porous carbon, (b) HRTEM image of Cu-N-C, (c) XRD patterns of porous carbon and Cu-N-C.


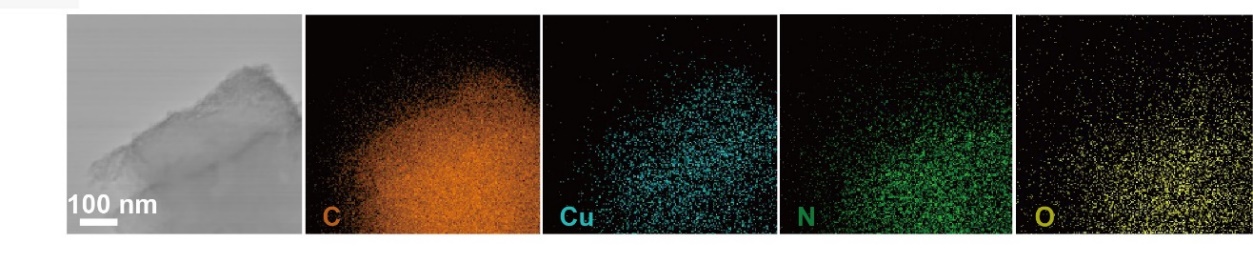


**Supplementary Fig. 5** Bright-field STEM image and corresponding EDS mapping images for Cu-N-C.


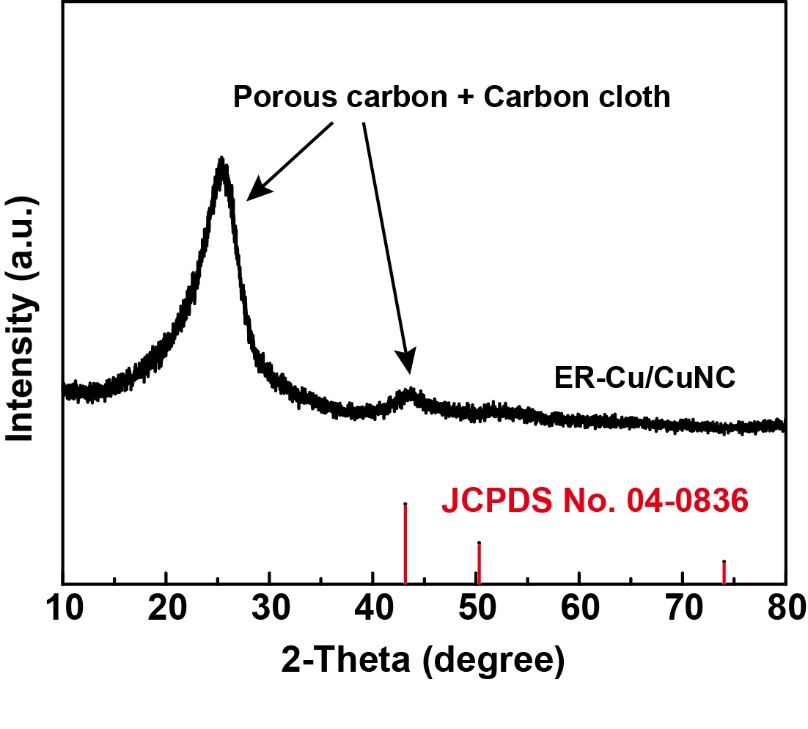


**Supplementary Fig. 6** XRD pattern of ER-Cu/CuNC.


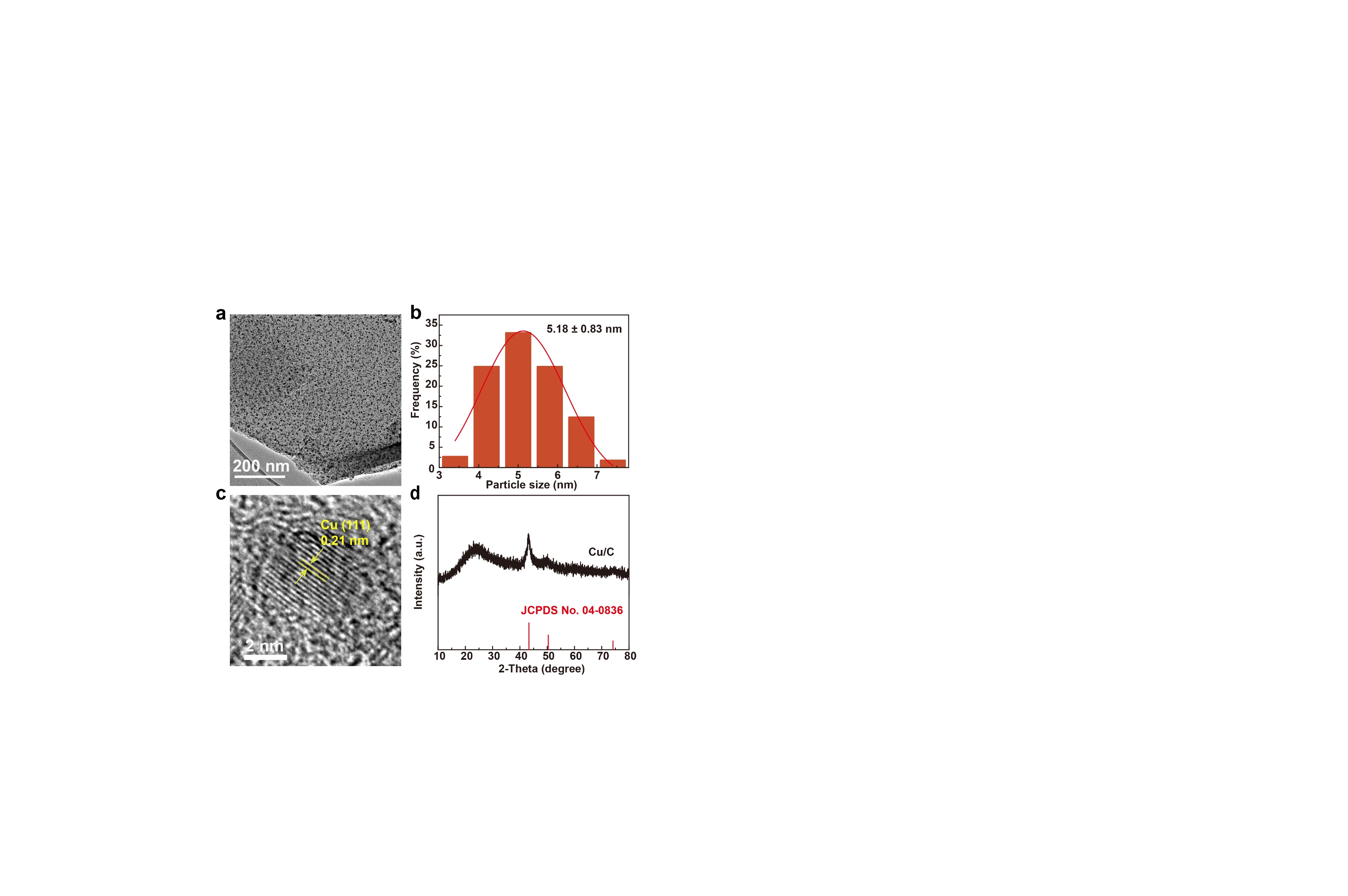


**Supplementary Fig. 7** (a) TEM image, (b) the particle size distribution, (c) HRTEM image, (d) XRD patterns of Cu/C.


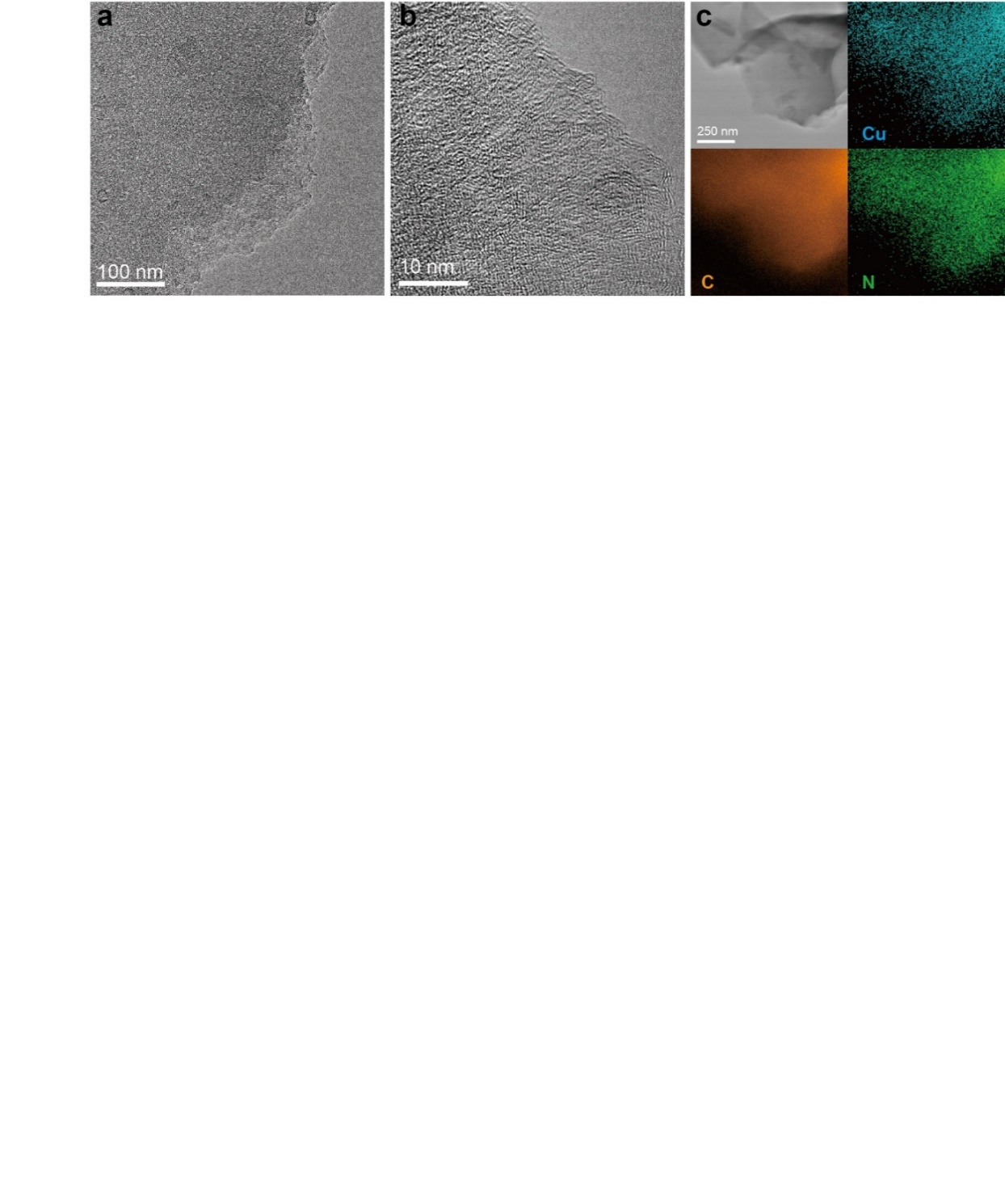


**Supplementary Fig. 8** (a) TEM image, (b) HETEM image, (c) EDS mapping images of CuPc/C.


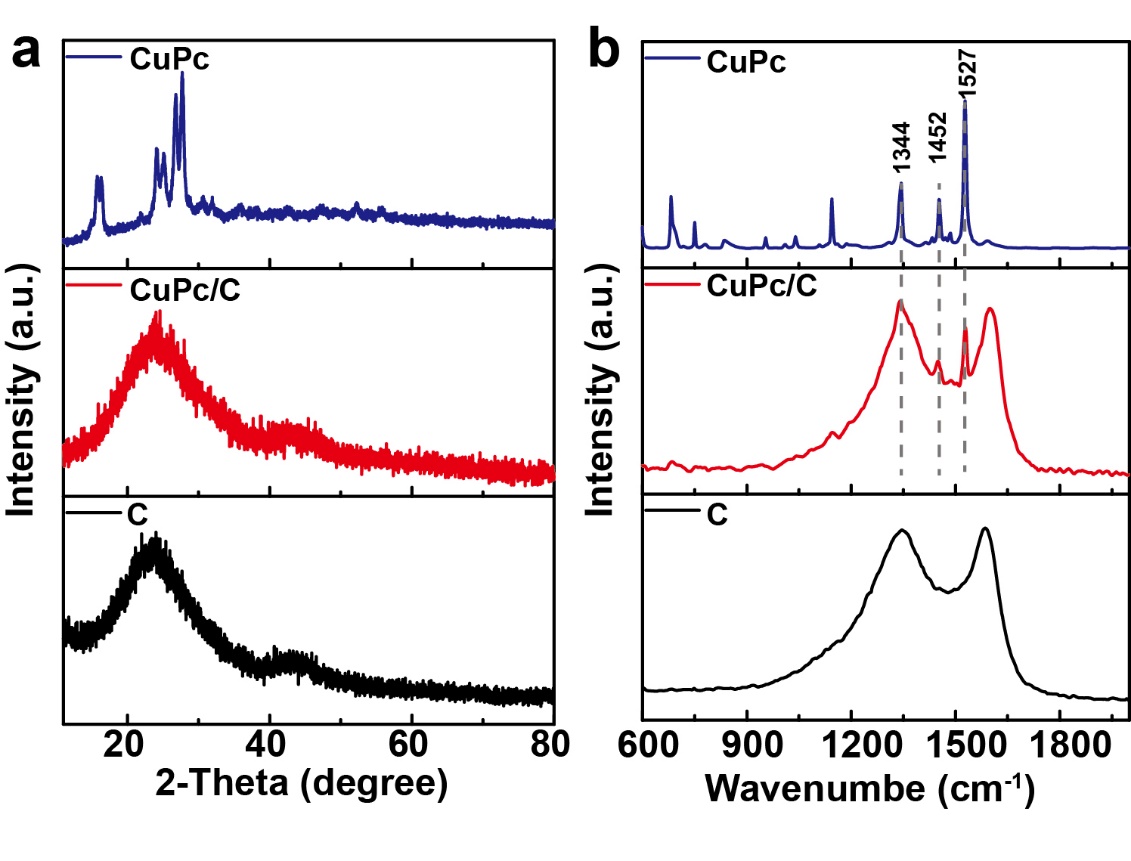


**Supplementary Fig. 9** (a) XRD patterns, (b) Raman patterns of C, CuPc/C, and CuPc.


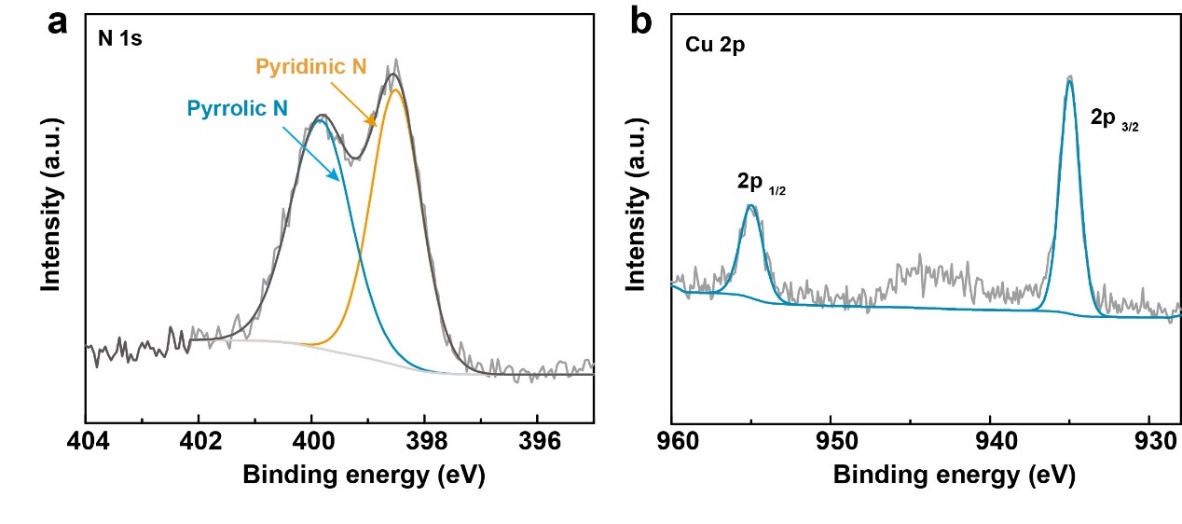


**Supplementary Fig. 10** (a) High-resolution N1s XPS spectra, (b) Cu 2p XPS spectra of CuPc/C.


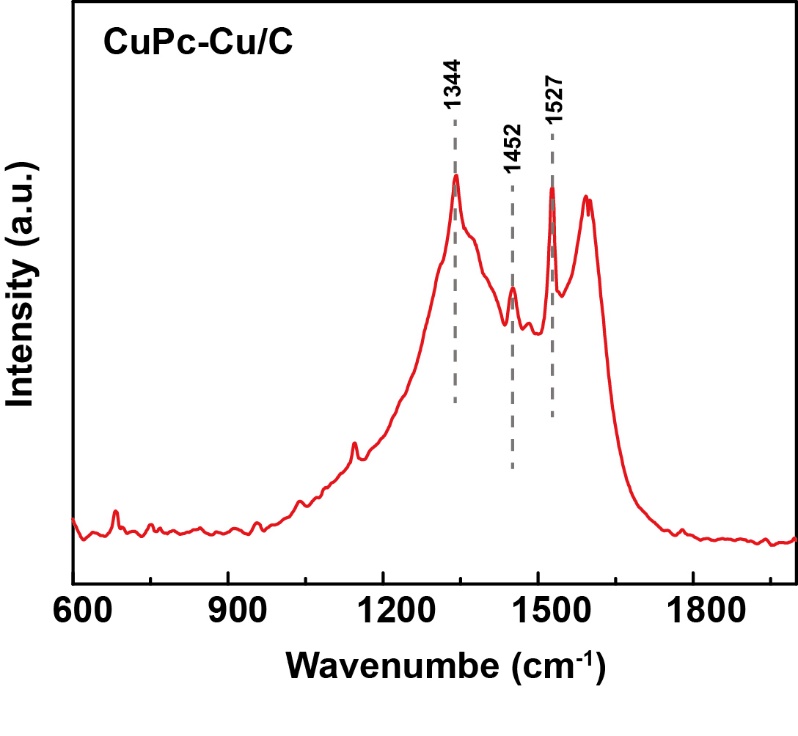


**Supplementary Fig. 11** Raman pattern of CuPc-Cu/C.


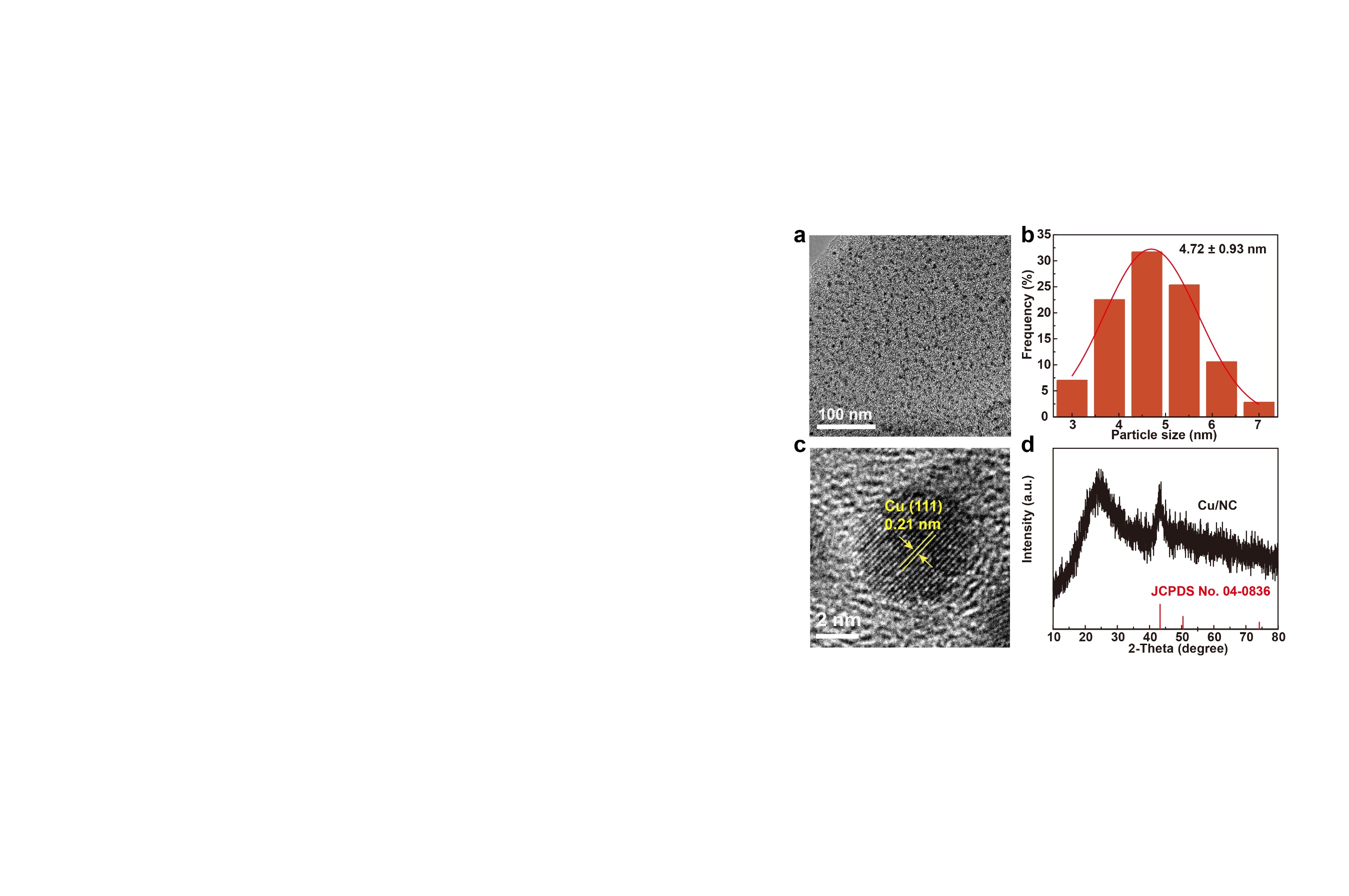


**Supplementary Fig. 12** (a) TEM image, (b) the particle size distribution, (c) HRTEM image, (d) XRD patterns of Cu/NC.


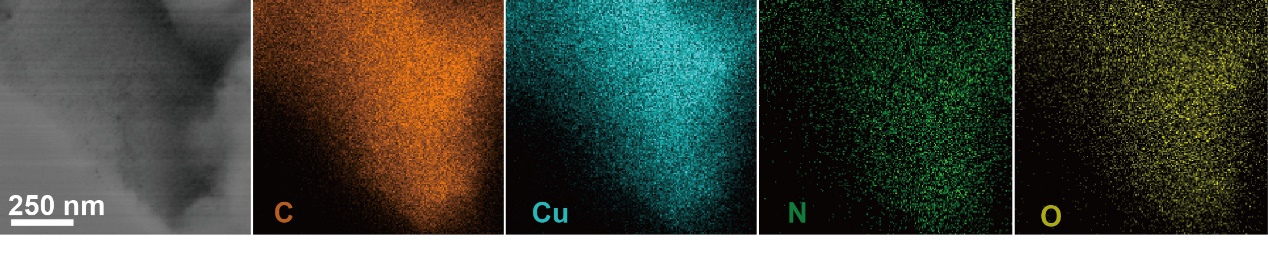


**Supplementary Fig. 13** The bright-field STEM and corresponding EDS mapping images of Cu/NC.


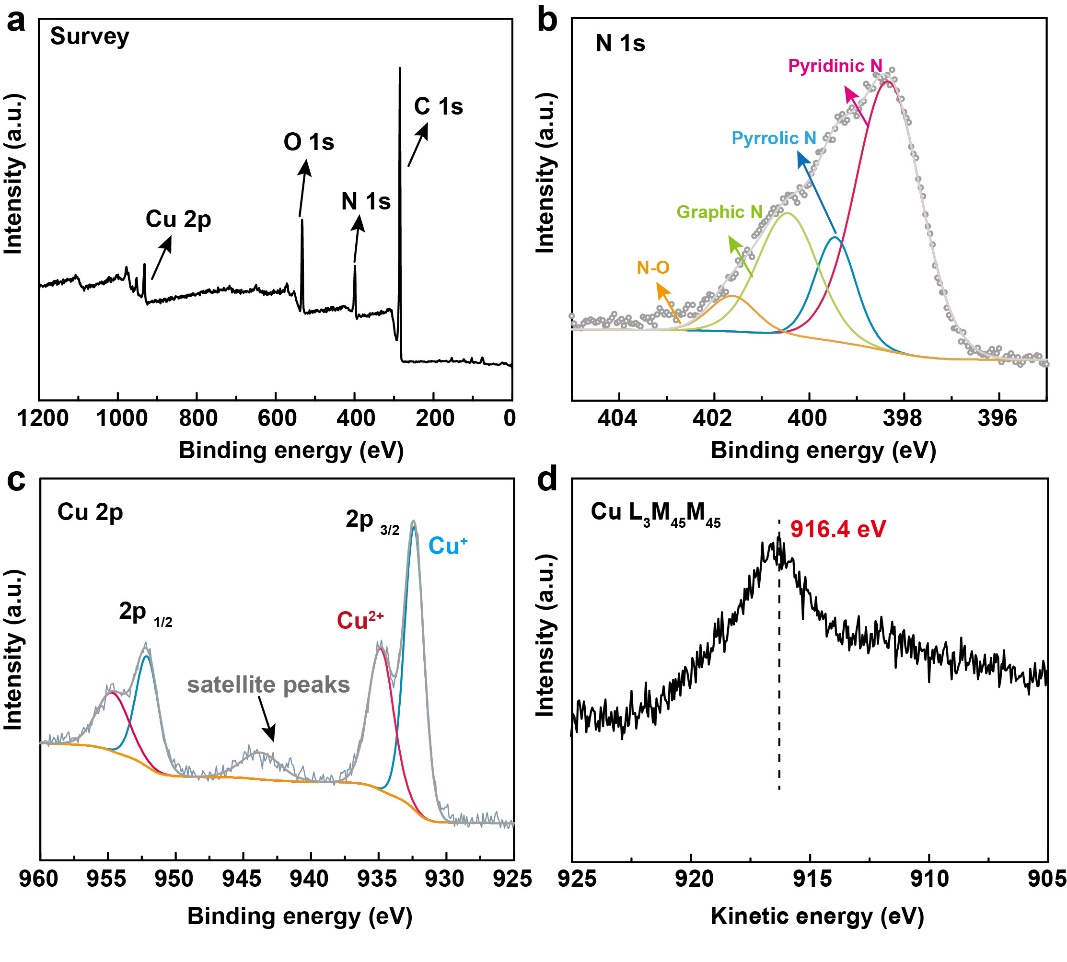


**Supplementary Fig. 14** (a)The survey XPS spectra, (b) the high-resolution N 1s XPS spectra, (c) the Cu 2p XPS spectra, and (d) the Cu Auger spectra of the Cu-N-C sample.


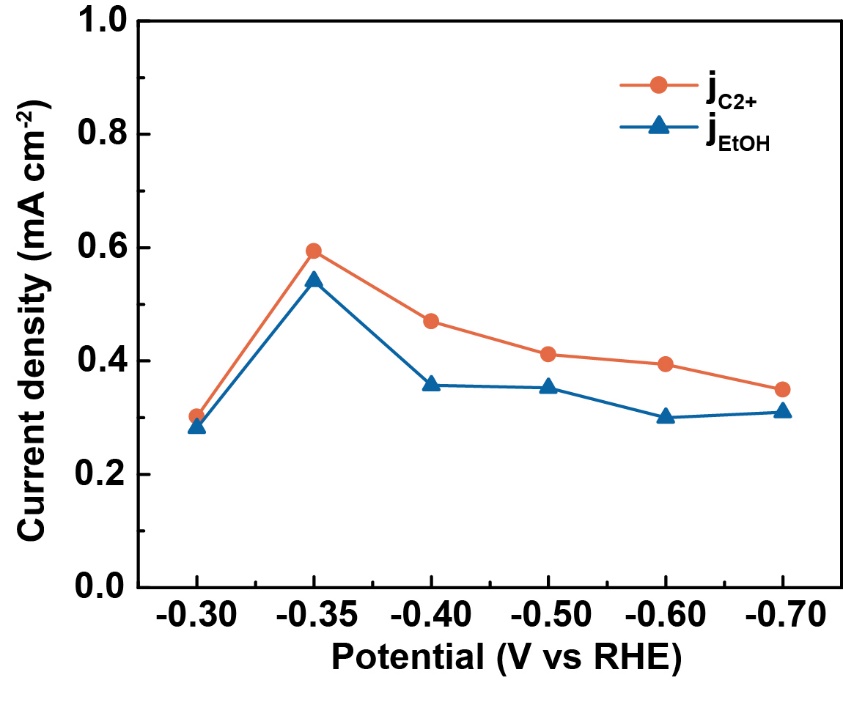


**Supplementary Fig. 15** Potential-dependent *j*_C2+_ and *j*_EtOH_ of the ER-Cu/CuNC catalyst for ECR.


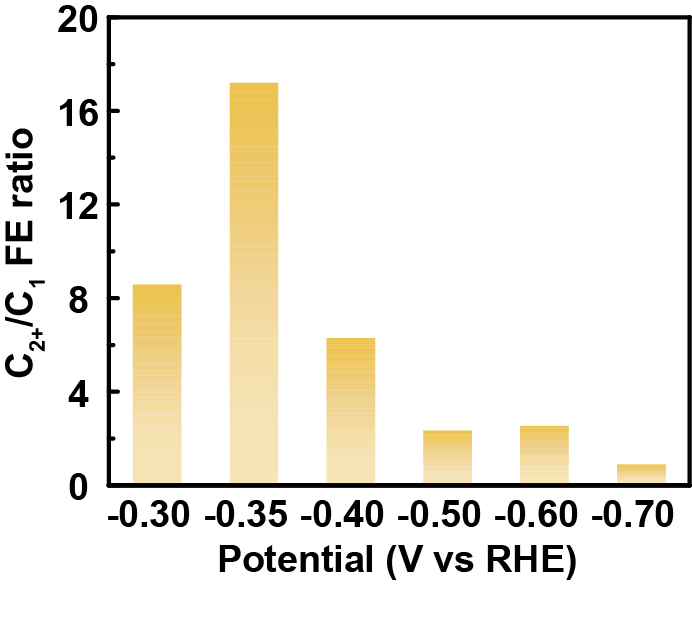


**Supplementary Fig. 16** The C_2+_/C_1_ FE radio in ECR products of the ER-Cu/CuNC catalyst at different potentials.


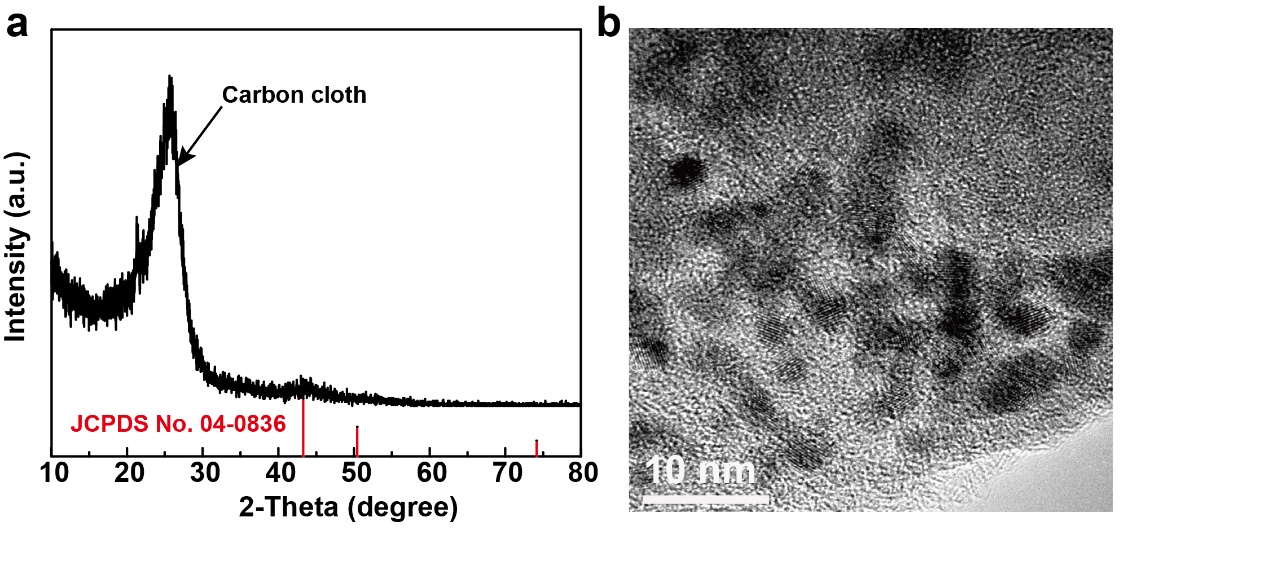


**Supplementary Fig. 17** (a) XRD pattern and (b) TEM image of ER-Cu/CuNC after ECR stability test.


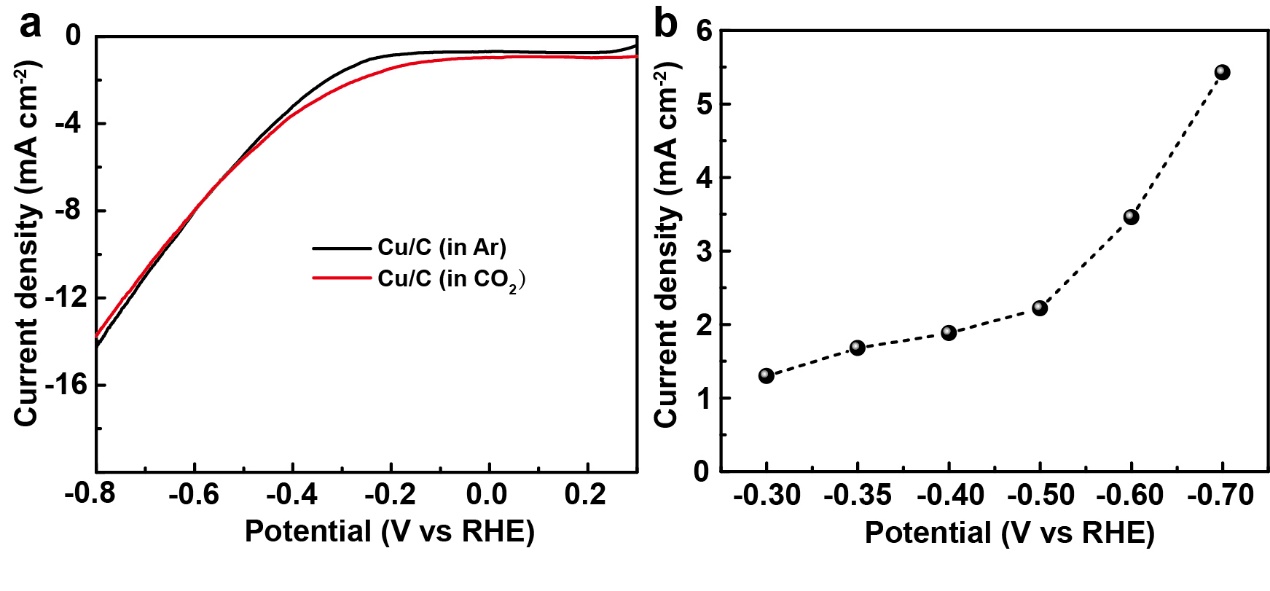


**Supplementary Fig. 18** (a) The LSV curves of the Cu/C catalyst performed in Ar-saturated and CO_2_-saturated 0.1 M KHCO_3_ solution, and (b) the potential-dependent current density.


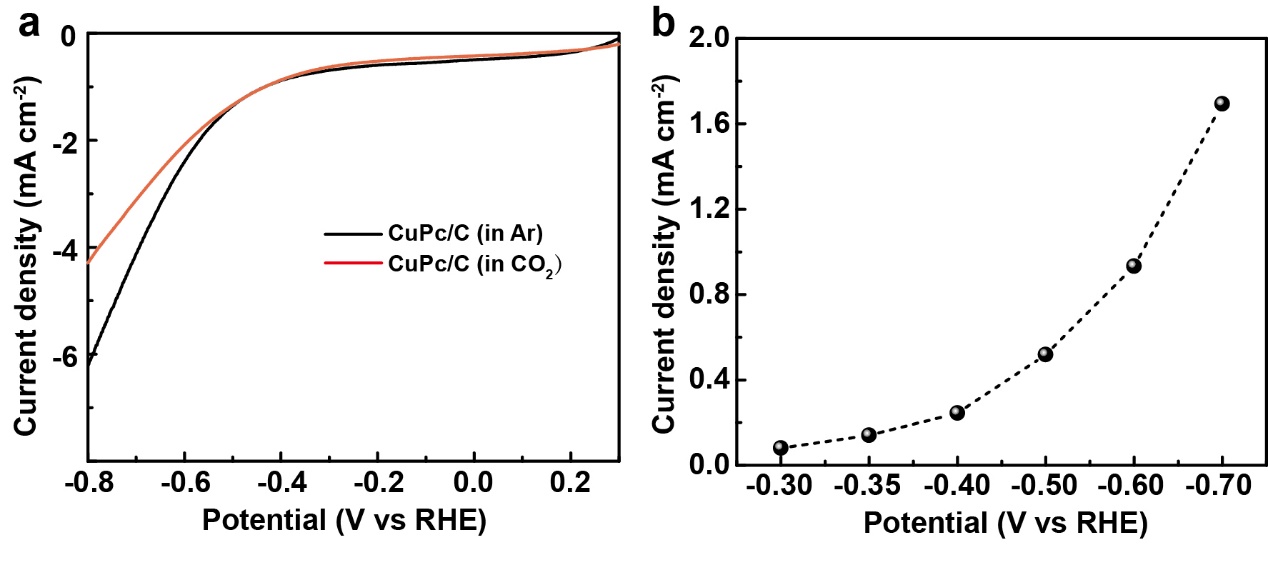


**Supplementary Fig. 19** (a) The LSV curves of the CuPc/C catalyst performed in Ar-saturated and CO_2_-saturated 0.1 M KHCO_3_ solution, and (b) the potential-dependent current density.


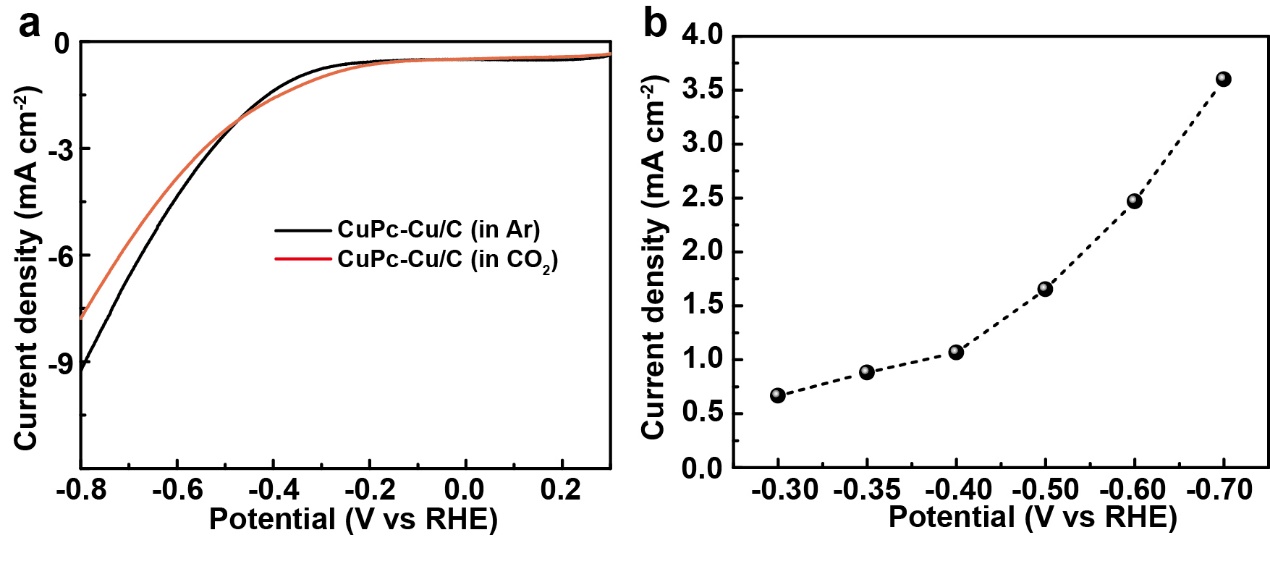


**Supplementary Fig. 20** (a) The LSV curves of the CuPc-Cu/C catalyst performed in Ar-saturated and CO_2_-saturated 0.1 M KHCO_3_ solution, and (b) the potential-dependent current density.


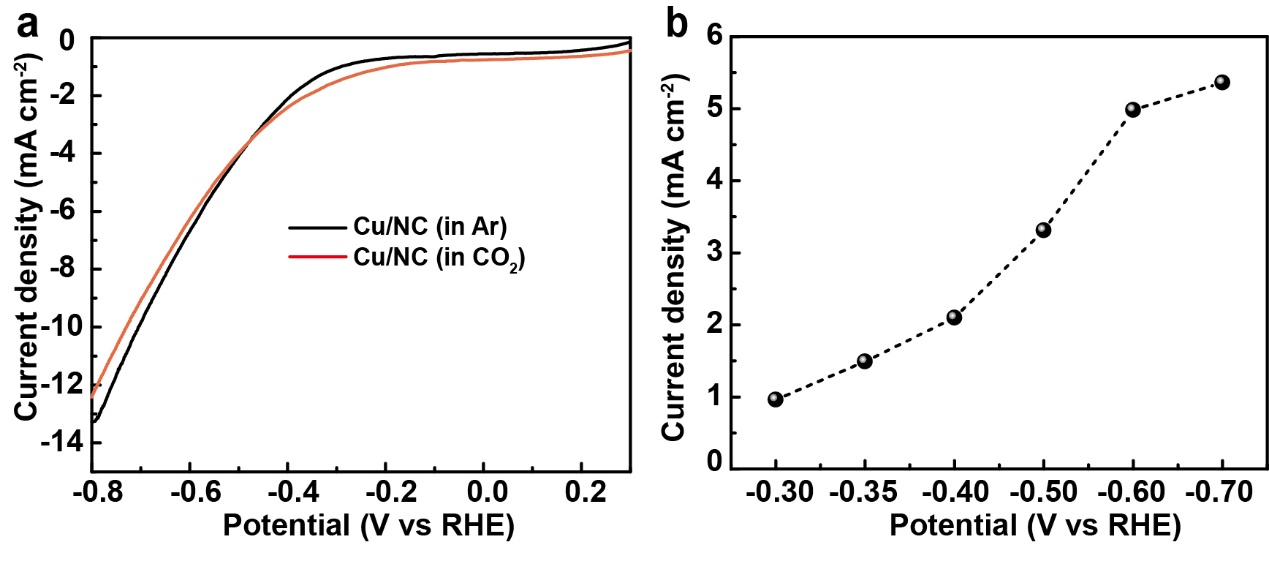


**Supplementary Fig. 21** (a) The LSV curves of the Cu/NC catalyst performed in Ar-saturated and CO_2_-saturated 0.1 M KHCO_3_ solution, and (b) the potential-dependent current density.


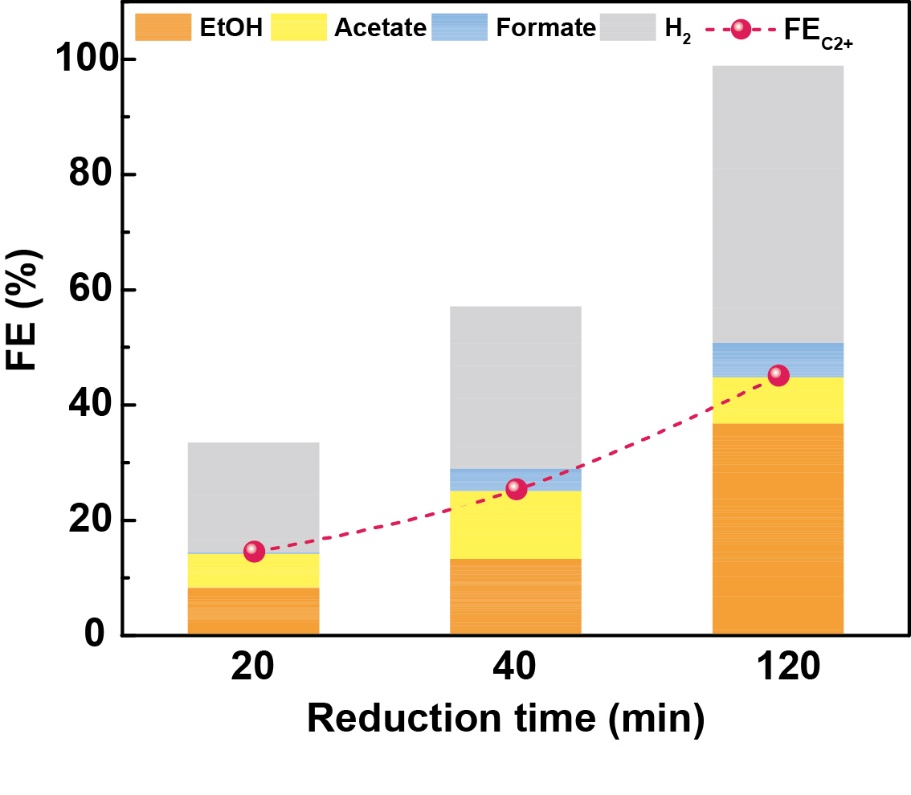


**Supplementary Fig. 22** Comparison of the selectivity of different products under different electrochemical reduction time at -0.30 V of Cu-N-C sample.

**Supplementary Table 1.** Elemental contents calculated from XPS results (atomic/%).

| Sample | C | N | O | Cu |
| --- | --- | --- | --- | --- |
| Cu-N-C | 78.26 | 9.99 | 10.63 | 1.12 |
| ER-Cu/CuNC | 86.01 | 4.14 | 8.9 | 0.95 |
| CuPc/C | 82.23 | 2.97 | 14.48 | 0.31 |
| Cu/C | 83.68 | 0 | 15.18 | 1.13 |
| CuPc-Cu/C | 80.73 | 5.12 | 12.88 | 1.28 |
| Cu/NC | 82.29 | 5.32 | 11.22 | 1.17 |

**Supplementary Table 2.** Comparison of recently reported Cu-based electrocatalysts for the electrochemical conversion of CO_2_ to ethanol.

| Catalyst | Electrolyte | Potential  (V vs. RHE) | Cell Type | FE_EtOH_  (%) | Ref |
| --- | --- | --- | --- | --- | --- |
| Cu/CuNC interface sites | 0.1 M KHCO_3_ | -0.35 | H-type cell | 55 | **This work** |
| 3D dendritic Cu-Cu_2_O | 0.1 M KCl | -0.40 | H-type cell | 32 | [1] |
| B-doped Cu-Zn GDE | 1 M KOH | -0.45 | H-type cell | 31 | [2] |
| Hierarchical Cu | 1 M KOH | -0.68 | Flow cell | 23.7 | [3] |
| Ag-doped Cu_2_O nanocube | 0.1 M KHCO_3_ | -0.98 | H-type cell | 17 | [4] |
| 3.6 µm Cu_2_O film | 0.1 M KHCO_3_ | -0.99 | H-type cell | 16.5 | [5] |
| Cu(100) with defects and Cu(I) species | 0.1 M KHCO_3_ | -1.00 | H-type cell | 32 | [6] |
| Cu SACs | 0.1 M KHCO_3_ | -0.70 | H-type cell | 91 | [7] |
| Cu-N-C | 0.1 M CsHCO_3_ | -1.20 | H-type cell | 55 | [8] |
| Ag_0.14_Cu_0.86_ alloy | 1 M KOH | -0.67 | Flow cell | 41.4 | [9] |
| Nanoporous Cu-Ag Alloy | 1 M KOH | -0.68 | Flow cell | 25 | [10] |

**REFERENCES**

1. Zhu Q, Sun X and Yang D *et al.* Carbon dioxide electroreduction to C_2_ products over copper-cuprous oxide derived from electrosynthesized copper complex. *Nat Commun* 2019; **10**: 3851.

2. Song Y, Junqueira J R C and Sikdar N *et al.* B-Cu-Zn gas diffusion electrodes for CO_2_ electroreduction to C_2+_ products at high current densities. *Angew Chem Int Ed* 2021; **60**: 9135-41.

3. Niu Z Z, Gao F Y and Zhang X L *et al.* Hierarchical copper with inherent hydrophobicity mitigates electrode flooding for high-rate CO_2_ electroreduction to multicarbon products. *J Am Chem Soc* 2021; **143**: 8011-21.

4. Herzog A, Bergmann A and Jeon H S *et al.* Operando investigation of Ag-decorated Cu_2_O nanocube catalysts with enhanced CO_2_ electroreduction toward liquid products. *Angew Chem Int Ed* 2021; **60**: 7426-35.

5. Ren D, Deng Y and Handoko A D *et al.* Selective electrochemical reduction of carbon dioxide to ethylene and ethanol on copper(I) oxide catalysts. *ACS Catal* 2015; **5**: 2814-21.

6. Arán-Ais R M, Scholten F and Kunze S *et al.* The role of in situ generated morphological motifs and Cu(I) species in C_2+_ product selectivity during CO_2_ pulsed electroreduction. *Nat Energy* 2020; **5**: 317-25.

7. Xu H, Rebollar D and He H *et al.* Highly selective electrocatalytic CO_2_ reduction to ethanol by metallic clusters dynamically formed from atomically dispersed copper. *Nat Energy* 2020; **5**: 623-32.

8. Karapinar D, Huan N T and Ranjbar Sahraie N *et al.* Electroreduction of CO_2_ on single-site copper-nitrogen-doped carbon material: Selective formation of ethanol and reversible restructuration of the metal sites. *Angew Chem Int Ed* 2019; **58**: 15098-103.

9. Li Y C, Wang Z and Yuan T *et al.* Binding site diversity promotes CO_2_ electroreduction to ethanol. *J Am Chem Soc* 2019; **141**: 8584-91.

10. Hoang T T H, Verma S and Ma S *et al.* Nanoporous copper–silver alloys by additive-controlled electrodeposition for the selective electroreduction of CO_2_ to ethylene and ethanol. *J Am Chem Soc* 2018; **140**: 5791-7.
